# The Dutch health care performance report: seven years of health care performance assessment in the Netherlands

**DOI:** 10.1186/1478-4505-12-1

**Published:** 2014-01-09

**Authors:** Michael J van den Berg, Dionne S Kringos, Lisanne K Marks, Niek S Klazinga

**Affiliations:** 1 National Institute for Public Health and the Environment (RIVM), PO Box 1, 3720 BA Bilthoven, The Netherlands; 2 Department of Social Medicine, Academic Medical Centre, University of Amsterdam, PO Box 22700, 1100 DE Amsterdam, The Netherlands

**Keywords:** Health care, Health system performance assessment, Performance indicators

## Abstract

In 2006, the first edition of a monitoring tool for the performance of the Dutch health care system was released: the Dutch Health Care Performance Report (DHCPR). The Netherlands was among the first countries in the world developing such a comprehensive tool for reporting performance on quality, access, and affordability of health care. The tool contains 125 performance indicators; the choice for specific indicators resulted from a dialogue between researchers and policy makers. In the ‘policy cycle’, the DHCPR can rationally be placed between evaluation (accountability) and agenda-setting (for strategic decision making). In this paper, we reflect on important lessons learned after seven years of health care system performance assessment. These lessons entail the importance of a good conceptual framework for health system performance assessment, the importance of repeated measurement, the strength of combining multiple perspectives (e.g., patient, professional, objective, subjective) on the same issue, the importance of a central role for the patients’ perspective in performance assessment, how to deal with the absence of data in relevant domains, the value of international benchmarking and the continuous exchange between researchers and policy makers.

## Background

Although the first examples of the assessment of health care systems performance may be traced back to centuries ago [1,2], the first attempts to systematically measure and compare performance of health care systems on a regular basis only started about fifteen years ago. The World Health Organization (WHO) describes Health Systems Performance Assessment (HSPA) as “a country-owned process that allows the health system to be assessed holistically, a ‘health check’ of the entire health system” [3]. Statistical indicators are used to monitor system performance. Although research on specific interventions, programs and sectors is of importance, the system-wide, holistic approach of HSPA has an important added value.

Most developed and transitional countries are facing similar challenges: an aging population, an increase in the prevalence of chronic illnesses, rising expenditures, inequity, etc. These issues require macro-level policy and, accordingly, information on how the system is functioning [4-6]. The ministerial conference in Tallinn in 2008 and the resulting “Tallinn Charter” has accelerated the HSPA-movement and marked the starting point for several countries [7]. The Tallinn Charter commits countries to produce measurable results and to promote transparency and accountability for their health care systems. The Tallinn Charter can be placed against the background of the governance shift in public administration towards ‘New Public Management’ (NPM). In the context of broader political and economic trends, NPM entailed the introduction of business-inspired concepts to the public administration to improve accountability. NPM promoted cost containment and stimulated the private sector to enter areas that were formerly reserved by the state, like the health sector. Consequently, health care system reforms and new health care system models became an important issue on the policy agenda of many countries. One of the characteristics of NPM is the greater focus on (especially quantitative) performance indicators [8]. This commitment also brings along an increased need for international comparisons. Creating an overview of the whole system fulfills an increasing need by policy-makers to be accountable to the public, the desire of policy-makers for better strategic planning to meet desired outcomes, and the mutual benefit of benchmarking for health care system re-engineering [9].

The Netherlands started to develop a performance framework for the Dutch health care system in 2002 [10], being one of the first countries in the world to do so. This resulted in the publication of the first Dutch Health Care Performance Report (DHCPR) in 2006 [11]. The report contains 125 performance indicators reporting on the quality, accessibility, and costs of the Dutch health care system. Seven years later, three editions of the DHCPR have been released in both Dutch and English. The Dutch website of the DHCPR presents all indicators and has several updates a year [12].

Given the relatively long experience with the assessment of the Dutch health care system which started long before the Tallinn conference, other countries may profit from lessons learned in the Netherlands. It was also for this reason, that the WHO recently organized an expert meeting in the Netherlands on health systems performance assessment which was attended by ministerial representatives from more than 19 countries of the WHO European Region, indicating the international interest in developing and improving health system performance assessments [13].

In this article we discuss the development process of the DHCPR including the conceptual approach of assessing the health care system, the role of the DHCPR in Dutch health policy, and important lessons learned in seven years of health care system performance monitoring. We intend to share our experiences and the lessons learned in developing this instrument rather than thoroughly evaluate this process.

## Development phases of the DHCPR

The DHCPR has been developed and is regularly evaluated and adapted in two phases [14].

### Phase 1: Development of conceptual framework

In cooperation between policy makers and researchers the main objectives for the DHCPR were defined. These objectives were i) to deliver policy relevant information to support priority setting and policy evaluation; ii) to deliver an overview of the performance of the Dutch health care system at system level using performance indicators, and iii) to identify gaps in the available knowledge and information on health care system performance. This required balancing policy objectives, the scientific state of the art, and actual possibilities of the use of data.

The starting point for the development of the indicator domains were the three health care policy objectives of the Dutch Ministry of Health (MoH), namely quality of care, accessibility, and affordability; this determined the broad focus of the DHCPR. The DHCPR indicates the performance of facilities and providers active in curative, long-term care and public health, covering all quality domains, at different aggregation levels. The DHCPR mainly focuses on health care rather than on public health. An important reason for this is that public health is already the focus of attention in another recurrent publication, the Dutch Public Health Status and Forecasts Report’ (see http://www.vtv2010.nl/english-editions/).

**Figure 1:**
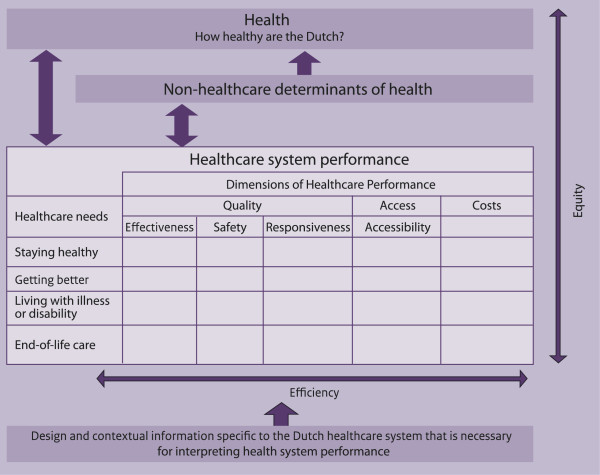
Conceptual framework of the DHCPR.

The conceptual framework was developed based on a systematic literature review of existing performance measurement systems, extensive consultations with (inter)national health care system experts and academics, conceptual analysis of the indicator domains (indicated in the matrix of Figure 1), and discussions with the MoH. An initial version of the conceptual framework was based on the Lalonde model and the balanced score card [15]; many of the indicator domains and definitions of that model are still used in the DHCPR nowadays. The framework has been further developed, combining parts of existing frameworks in other countries. For instance the basic health needs from de Agency for Health care Research and Quality framework were included. Figure 1 shows the conceptual framework of the DHCPR in its current form. It maintains a broad perspective on health and its determinants, and recognizes the key aims of health policy in the Netherlands. The framework has been described in detail by Arah et al. [16].

### Phase 2: Indicator selection

**Figure 2:**
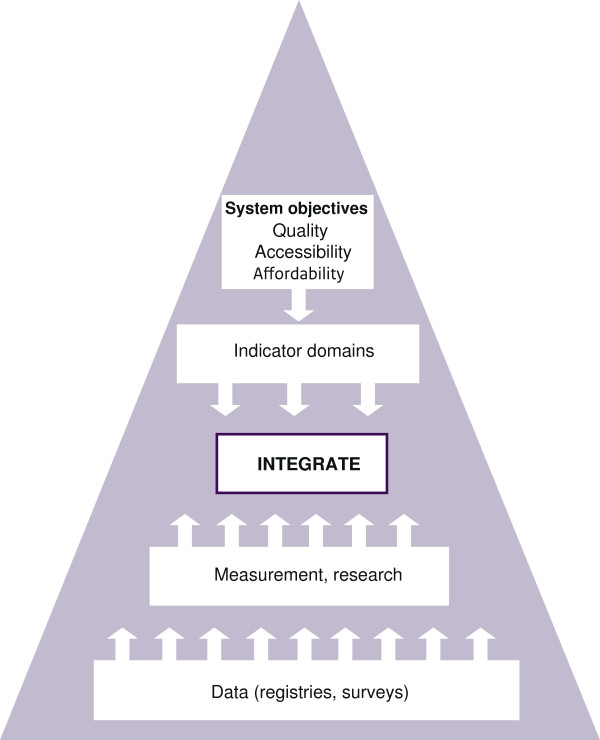
Top-down and bottom-up indicator selection process of the DHCPR.

Figure 2 summarizes the process of indicator selection. The DHCPR aims to fill the conceptual framework with the most useful indicators of health care performance that give the most complete indication of real practice. Starting point for the selection of data sources to populate indicators is to use available data. It was a specific request by the MoH in 2006 to avoid the need to set up new data collections and registries.

We can distinguish five types of data sources ‘feeding’ the DHCPR:

1. Population and patient surveys on their health care experiences (with health care insurers, facilities and providers);

2. Provider surveys on the (potential) quality of their services delivery;

3. Clinical registries and administrative databases kept by health care providers, facilities, and insurers on the services provided and characteristics of their population/patients;

4. Surveys and annual reports by health care facilities and insurers on their financial and human resources;

5. Geographical access information on the location of health care providers and facilities.

The selection of the indicators was a result from balancing the top-down approach with the bottom-up approach. From the top the health care system’s objectives determine the indicator domains and relevant indicators to be used, while at the bottom the data sources and scientific state of the art determine the data availability and reliability to populate indicators.

Therefore, the final selection of indicators is often a compromise between the conceptual relevance and the practical possibilities. The composition of indicators of the DHCPR has been changed at times due to new scientific insights, changing policy priorities, or public attention for certain topics, giving rise to a need for monitoring certain health care system performance aspects. Currently, a list of 125 indicators is used to cover the needs (the rows) in the framework and the system goals (the columns). The full list of indicators can be found in appendix 2 of the report [17].

## Position in the policy process

**Figure 3:**
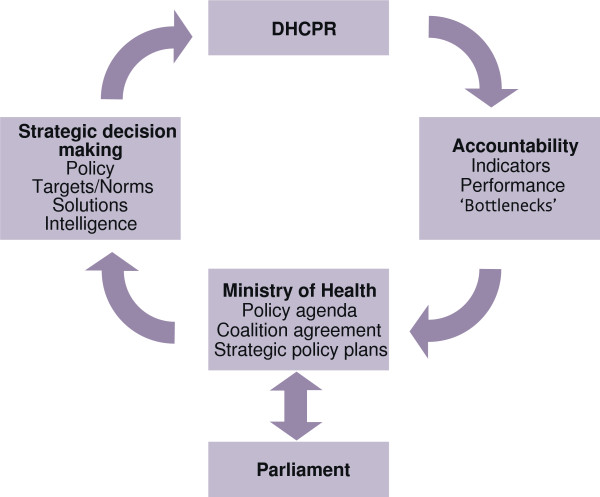
Place of DHCPR in the policy cycle.

The former Dutch Minister of Health characterized the report as *“a solid empirical foundation for the policy of the Ministry of Health”*[17]. Like other HSPA-reports, the DHCPR is fulfilling several functions in the rational model of policymaking: agenda-setting (problem recognition), policy formulation (proposal of solutions), decision-making (choice of solution), policy implementation (putting solution into practice), and policy evaluation (monitoring the results) [18]. The DHCPR can ‘rationally’ be placed between evaluation (accountability) and agenda-setting (for strategic decision-making). Figure 3 shows the different functions and the position of the DHCPR in the policy cycle.

The DHCPR measures health care performance in three domains using a set of indicators and formulates, so called key messages, about what aspects of the health care system went well, the aspects that went less well, and what requires attention. This information is reported to the minister and the parliament and can be used for priority setting, and the formulation of policy. In return, policy decisions, proposed solutions, and eventually policy targets, are used as new input for the DHCPR, which monitors the progress on these issues. The conclusions of the DHCPR are presented in an accessible manner and are easily available for the general public. We briefly explain how the DHCPR fulfills this role with regard to agenda-setting and accountability.

## Agenda-setting for strategic decision-making

The aim of the DHCPR is to make a contribution to the strategic decision-making of the MoH in the area of health care. To realize that, there are two more specific objectives:

First, the DHCPR attempts to paint a broad picture of the performance of the health care system. To do so, it presents trends over time, compares the Netherlands to other countries and, where possible, states (policy) standards and benchmark data. It provides policymakers the information they need to make their own assessment of the performance of the Dutch health care system. The DHCPR is typically useful for periodical milestones in policy such as the formulation of a coalition agreement (every 4 years), formulation of the policy agenda (annually in September), and the National Budget (annually in September).

The second function may be referred to as data intelligence; by integrating information on performance from other sources, the DHCPR is a signpost towards an enormous amount of data sources available in the country. The DHCPR can guide the way through the sometimes diffuse world of performance information. By also signaling which essential information is lacking, the DHCPR provides input for the research and development agenda to strengthen the national information infrastructure.

## Accountability

Although health care providers are the first responsible for the quality of care provided, the minister has a ‘system-responsibility’; she is primarily responsible for a good functioning of the system as a whole including the conditions for high quality care, accessibility for all, and the efficient use of resources. As is mentioned in the Tallinn Charter, ministries commit themselves to be accountable for system performance and to achieve measurable results.

The DHCPR is also used by the minister as a regular update on the state of affairs for parliament; the parliament, in turn, may ask questions to the minister. Although accountability is an ongoing process, there is also an annual milestone which is called Accountability day. On the third Wednesday in May, all ministers present their annual reports stating their achievements and activities, and related costs.

## Lessons learned

The DHCPR has resulted in a range of key messages about the Dutch health care system. For a detailed description of these results we refer to the reports that can be found at the website (www.healthcareperformance.nl). Here, we will only mention a few and highlight three important overall conclusions. Next, we will sum up some of the key lessons learned about the process of measuring performance systematically and developing the DHCPR.

### About the Dutch health care system

Accessibility is one of the strongest points of the Dutch health care system. Compared to many other countries, most services are within easy reach. Around 99% of the population can reach a general practitioner and pharmacy within ten minutes by car. Reaching the nearest hospital takes less than 30 minutes. The system is also accessible from a financial perspective; there is a broad basic benefits package under which practically all residents of the country are insured for health care costs. Co-payments are amongst the lowest in the OECD countries. Compared to other countries, very few people forego care because of financial reasons. The well-organized system reflects a tradition of formalized solidarity, as mentioned by Westert et al. [19], “The Dutch live below sea level behind dykes, and history has taught them that solidarity pays off. This solidarity has built a healthcare system that treats all alike”. However, a clear view on those who are really worst off, such as illegal immigrants and homeless people, is still lacking. The increase of the yearly mandatory deductible from 220 Euro to 350 Euro may affect financial access for some people.

Health care expenditures have increased spectacularly in the past decades. In the DHCPR, it was reported that the Netherlands spent 9% of the GDP on health care, which was around the average of the Western European countries. Currently, the Netherlands is the biggest spender in Europe and the second (after the US) among the OECD countries. In 2011, the expenditures were estimated at 12% of the GDP according to the system of health accounts. The high and rising expenditures are the most important challenge for Dutch policy makers. Examples of measures that are introduced to manage this are the increase of deductibles and a large reform of the Exceptional Medical Expenses Act, which covers mainly long-term care and care for the disabled.

In 2006, the system for the financing of curative care was changed and the Health Insurance Act came into force. A managed competition model was introduced in which health insurers play a central role; health insurance companies were expected to act more as contracting parties, demanding effective, high quality services. By strengthening the role of insurers and improving the freedom of choice for patients, this system reform was meant to guarantee sustainability, quality, and efficiency of the system. An analysis of the effects of the system reform until 2010 showed that very few of the desired effects had been achieved. No substantial changes in quality and access have been recorded since the reform and trends were comparable with neighboring countries. Good information about quality on which patients and insurers can base their choices is still scarce and so far quality of care has played only a minor role in negotiations between health care providers and insurers. This information for patients and health insurers requires a far more detailed level than the type of information provided in the DHCPR. Rather than saying something about the system, such information should show differences in performance between providers. Macro costs have been rising more rapidly after 2006, due to, among other causes, the shift of previously privately insured persons towards the mandatory public insurance.

### About health systems performance assessment: seven recommendations

Based on our experience in the Netherlands, we have identified seven recommendations that are essential to the development, process, and/or outcome of HSPA.

First, anyone who wants to design a tool for the overall assessment of a health care system has to deal with the question of how the system should be conceptualized: what aspects should be included and which dimensions, areas, domains, concepts, etc., can be distinguished within the system. In the literature, a range of frameworks is reported; some of them are designed for international comparisons, others for the assessment of one specific system. Designing a framework is no mathematics and will never be the result of just a scientific enterprise. The DHCPR framework is the product of an extensive review of the performance literature on the one hand and extensive exchange of ideas and needs of policy makers at the MoH on the other. Realizing the complex and dynamic characteristics of policy making, this interaction aims to contribute to a higher relevance of the DHCPR for policy makers. This discussion is very useful because it forces all parties to explicate what is really important. A possible pitfall is that ‘framework discussions’ can easily end up in rather abstract philosophical discussions that distract the participants from the real performance measurement.

Second, much information about performance can be found in one-off studies. Even when these studies are scientifically sound and relevant, it should be taken into account that repeated measurement is an important criterion for structural performance assessment. Statistics become more relevant for policy makers when developments over time can be followed and possible effects of policy measures can be shown. However, some one-off studies may be very relevant and, in such cases, performance reports may plead for a follow-up.

Third, an important added value of performance reports such as the DHCPR is that they combine multiple perspectives on the same problem. For example, in the DHCPR the problem of the shortage of manpower in some sectors is presented by:

– The number of hard-to-fill-vacancies in healthcare (perspective of employers)

– Percentage of personnel leaving the sector (turnover)

– Percentage of work hours lost (absenteeism)

– Percentage of care users who believe sufficient personnel is available during a stay in hospital or nursing home

– Number of doctors and nurses per 1,000 population (as a contextual indicator)

Also, when existing information of secondary sources is used, the combination of perspectives (e.g., objective and subjective, employers’ and patients’ perspectives) may lead to new conclusions [20].

Fourth, and in addition to the third point, patient experiences play a central role in the DHCPR and should play a role in health care system performance instruments in general. In the first edition, patient experiences were defined as a separate indicator domain. In subsequent editions, it was decided to include patient perspective indicators in several other domains. For instance, patient experiences with safety, effectiveness, financial access, freedom of choice, and timeliness were covered. Instead of being concentrated in a separate domain, the patient became one of the common threads in the report. Taking patient experiences into account is part of a broader debate on how to measure quality in health care: who decides what quality is? There is an increasing awareness that ‘objective’ outcomes are not enough, but that the way people (or patients) experience health care is also essential. With the move towards managed competition in health care, patients are now considered as active consumers of health care with a greater responsibility in making health care choices. Moreover, patients deliver another kind of ‘expertise’ additional to the medical professionals’ traditional focus on what can be valuable to improve the quality of health care. The DHCPR makes use of the Dutch Consumer Quality indices. This is a standardized method that includes the consumer’s perspective (including both experiences and what they consider important in health care) for comparing the performance of health care providers. In light of the need for transparency of information in the present health care system and the system’s accountability, the inclusion of patient experiences in the DHCPR is essential.

**Figure 4:**
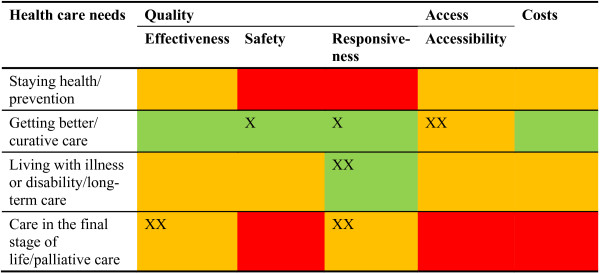
**Availability of empirical data for the indicator domains.** Green = good; Orange = moderate; Red = poor. X: improved between 1^st^ and 2^nd^ DHCPR; XX: improved between 2^nd^ and 3^rd^ DHCPR.

Fifth, the choice of relevant indicator domains should not be avoided because of a lack of available data. Besides presenting a wealth of information, the DHCPR also reviews critically to what extent the framework can be filled with indicators. Sometimes, this reveals essential data caveats. Figure 4 shows the extent to which the DHCPR was able to fill relevant domains in the period that is covered by the first three editions of the report. Previously in this article, we mentioned that in 2006 the MoH requested to avoid the need to set up new data collections and registries. Nevertheless, to draw a complete picture, investments in new data sources are still required. Ideally, the government bears accountability for the system in its full width, which requires information about all parts. Of course this need has to be balanced against an eventual increase of workload among health care providers due to registration. This matrix may be used for priority setting in research and development work.

Sixth, as mentioned in our introduction, the HSPA is a country-owned process. It is, however, important to be aware of the fact that there is a lot of performance measurement taking place both nationally and internationally. National initiatives can take advantage of such networks and initiatives that build up experience. The DHCPR is connected to several international networks, such as the OECD Health Data, and exchanges experiences with several other countries. Organizations like the WHO, OECD, and the European Observatory on Health Systems and Policies contributed largely to the body of knowledge on the HSPA from which we can profit [21].

Seventh, continuous exchange between researchers and policy makers is essential. Theoretically, the exchange between information and policy making can nicely be displayed using policy cycles (which we did in this article). However, creating policy impact remains a real challenge, as supported by extensive literature on policy-research relations [22-24]. While performance reports are mostly the product of a very thorough, rational, and long process, policy makers are often confronted with a rapidly changing political context. Governments and ministers come and go more often than foreseen and political debates are often dominated by the headlines on the front page of today’s newspaper. In the upcoming years, the DHCPR will try to add a more flexible part for which policy makers can suggest indicators that may be followed for a shorter period on top of the stable set.

The seven essential ingredients for the HSPA, based on our recommendations, are summarized as follows:

– Good conceptual framework

– Repeated measurements

– Multiple perspectives on the same problem

– Patients’ experiences as a common thread

– Way to signalize data caveats

– International network and perspective

– Continuous exchange between researchers and policy makers

## Towards the next DHCPR

Until 2013, three editions of the DHCPR have been released, in 2006, 2008, and 2010. After 2010, information has been updated twice a year on the website http://htttp://www.gezondheidsZorgbalans.nl. Starting in 2014, the report will be published once every four years. The reports are also published in English, see http://www.healthcareperformance.nl.

The next translated edition is expected at the end of 2014.

## Abbreviations

DHCPR: Dutch Health Care Performance Report; HSPA: Health Systems Performance Assessment; MoH: Ministry of Health; NPM: New public management; WHO: World Health Organization.

## Competing interests

The authors declare that they have no competing interests.

## Authors’ contributions

MJvdB was involved in the original idea and took the lead role in drafting the paper. DSK, LM and NSK contributed to the writing and critical revision of the paper. All authors read and approved the final manuscript.

## Authors’ information

MJvdB is a post-doctoral researcher at RIVM and AMC, and current project leader of the DHCPR; he contributed to all editions of the DHCPR. DSK is a post-doctoral researcher at AMC and an expert in the field of HSPA. LM is a master’s student and investigated the construction process of the DHCPR (2010) and its use in the policy process of the Dutch Ministry of Health. NSK is a professor at AMC and the head of the Health Care Quality Indicator program at the OECD. He was involved in the development of the DHCPR from the beginning and is currently a member of the scientific advisory committee of the DHCPR. He supervised several PhD projects that contributed to the development of the DHCPR.

## References

[B1] McIntyre D Rogers L Heier EJ Overview, history, and objectives of performance measurement Health Care Fin Rev 2001 22 7 21 PMC4194707 25372047

[B2] Smith PC Mossialos E Papanicolas I Leatherman S Performance Measurement for Health Systems Improvement experiences, challenges and prospects 2009 New York: Cambridge university press

[B3] World Health Organisation Regional Office for Europe Performance Assessment: A Tool for Health Governance in the 21st Century 2012 Copenhagen: WHO

[B4] Organization for Economic Co-operation and Development Health at a Glance: Europe 2012 2012 Paris: OECD

[B5] Walshe K McKee M McCarthy M Groenewegen P Hansen J Figueras J Ricciardi W European health management health systems and policy research in Europe: Horizon 2020 Lancet 2013 382 9893 668 669 10.1016/S0140-6736(12)62195-3 23515143

[B6] Klazinga NS Fischer C Ten Asbroek A Health services research related to performance indicators and benchmarking in Europe J Health Serv Res Policy 2011 16 Suppl 2 38 47 10.1258/jhsrp.2011.011042 21737528

[B7] World Health Organization Regional Office for Europe The Tallinn Charter: Health Systems for Health and Wealth 2008 Copenhagen: WHO

[B8] Hood C A public management for all seasons? Public Administration 1991 69 3 19 10.1111/j.1467-9299.1991.tb00779.x

[B9] Veillard JHM Performance Management in Health Systems and Services Studies on its Development and Use at International, National/Jurisdictional, and Hospital Levels 2012 Amsterdam: University of Amsterdam

[B10] Delnoij DMJ Ten Asbroek AHA Arah OA Custers T Klazinga NS Bakens zetten. Naar een Nederlands raamwerk van prestatie-indicatoren voor de gezondheidszorg 2002 Den Haag: Ministerie van VWS

[B11] Westert GP Verkleij H Dutch Health Care Performance Report 2006 2006 RIVM: Bilthoven

[B12] 2013 RIVM: De Zorgbalans [http://bit.ly/1hYwvcH] (In Dutch)

[B13] World Health Organization Regional Office for Europe Scope and Purpose. Meeting on Health Systems Performance Assessment for Health Governance. Bilthoven, The Netherlands. 16–17 February 2012 2011 Copenhagen: WHO

[B14] Van den Berg MJ Deuning C Gijsen R Hayen A Heijink R Kooistra M Lambooij M Limburg LCM Definitierapport Zorgbalans 2011 RIVM: Bilthoven

[B15] Ten Asbroek AHA Arah OA Geelhoed J Custers T Delnoij DMJ Klazinga NS Developing a national performance indicator framework for the Dutch health system Int J Qual Health Care 2004 16 Suppl 1 i65 i71 15059989 10.1093/intqhc/mzh020

[B16] Arah OA Klazinga NS Delnoij DM Ten Asbroek AH Custers T Conceptual frameworks for health systems performance: a quest for effectiveness, quality, and improvement Int J Qual Health Care 2003 15 377 398 10.1093/intqhc/mzg049 14527982

[B17] Westert GP Van den Berg MJ Zwakhals SLN De Jong JD Verkleij H Dutch Health Care Performance Report 2010 2010 Bilthoven: RIVM http://www.gezondheidszorgbalans.nl/object_binary/o10229_DHCPR-2010(def)[1].pdf

[B18] Howlett M Ramesh M Studying Public Policy: Policy Cycles and Policy Subsystems 2003 2 Oxford: Oxford University Press

[B19] Westert GP Burgers JS Verkleij H The Netherlands: regulated competition behind the dykes? BMJ 2009 339 b3397 10.1136/bmj.b3397 19736283

[B20] Nutley SM Walter I Davies HTO Using Evidence. How Research can inform Public Services 2007 Bristol: Policy Press

[B21] Forde I Morgan D Klazinga NS Resolving the challenges in the international comparison of health systems: the must do’s and the trade-offs Health Policy 2013 112 1–2 4 823 23434265 10.1016/j.healthpol.2013.01.018

[B22] Black N Evidence Based Policy: Proceed with Care BMJ 2001 323 7307 275 278 10.1136/bmj.323.7307.275 11485961 PMC1120888

[B23] Landry R Lamari M Amara N The extent and determinants of the utilization of university research in government agencies Public Admin Rev 2003 63 2 192 205 10.1111/1540-6210.00279

[B24] Lomas J Connecting Research and Policy Isuma: Canadian Journal of Policy Research 2000 1 1 140 144

